# Feeding Ecology of Wild Brown-Nosed Coatis and Garbage Exploration: A Study in Two Ecological Parks

**DOI:** 10.3390/ani11082412

**Published:** 2021-08-16

**Authors:** Delma Henriques Rodrigues, Eduardo Calixto, Clarice Silva Cesario, Renata Barcelos Repoles, Waldomiro de Paula Lopes, Viviane Silva Oliveira, Alessandro Brinati, Nadja Simbera Hemetrio, Ita Oliveira Silva, Vanner Boere

**Affiliations:** 1 Programa de Pós-graduação em Biologia Animal, Universidade Federal de Viçosa, Viçosa 36570-900, MG, Brazil; delmahenrique@gmail.com (D.H.R.); clair.fevasf@gmail.com (C.S.C.); repoles_bio@hotmail.com (R.B.R.); alexesperafeliz@yahoo.com.br (A.B.); itabio@hotmail.com (I.O.S.); 2 Programa de Pós-graduação em Entomologia, Universidade de São Paulo, São Paulo 05508-220, SP, Brazil; calixtos.edu@gmail.com; 3 Instituto Chico Mendes de Biodiversidade, PNC, Alto Caparaó 36834-000, MG, Brazil; waldomiro.lopes@gmail.com; 4 Ciências Biológicas, Universidade do Estado de Minas Gerais, Caratinga 35309-899, MG, Brazil; vivianeesperafeliz@yahoo.com.br; 5 Fundação de Parques Municipais e Zoobotânica de Belo Horizonte, Belo Horizonte 31210-090, MG, Brazil; nadjasimbera@gmail.com; 6 Instituto de Humanidades, Artes e Ciências, Universidade Federal do Sul da Bahia, BR 415, Sn, Itabuna 45660-000, BA, Brazil

**Keywords:** ecotourism, human–animal interactions, mammals, parks, *Nasua nasua*

## Abstract

**Simple Summary:**

Coatis are mammals that frequently exploit human food sources, such as dumps in ecological parks. This behavior can lead to changes in food ecology and health problems. To verify the change in the diet of wild coatis, 56 fecal samples were analyzed in two ecological parks visited by tourists, Parque Municipal das Mangabeiras (PMM) and Parque Nacional do Caparaó (PNC). Multivariate statistics were applied to evaluate the interactions among four variables (volume, composition, place, and sex of coatis). A significant interaction between parks and sexes with regard to volume and food category was not found. A decreasing gradient in volume was found in PNC males, followed by the PNC females, PMM males, and PMM females. No differences were found in categories of food between males and females from PNC and PMM, except for invertebrates, as females from PNC consumed more invertebrates than individuals from PMM. The coatis of both parks primarily consume invertebrates and vegetables, but garbage residues were found in feces. Human food and garbage fragments change feeding ecology. Garbage residues cause risks to the health of coatis. These findings suggest a problem to be addressed in efforts to preserve wild coatis in both parks.

**Abstract:**

Wild animals that feed on garbage waste are a problem in ecological parks as it can substantially alter their food ecology. Wild coatis that occupy human recreation areas in parks are often observed feeding on garbage, but the ecological consequences are scarcely known. Forty-four fecal samples from females and 12 from males of wild coatis living in two ecological parks (Parque Municipal das Mangabeiras (PMM) and Parque Nacional do Caparaó (PNC)) were analyzed. Multivariate statistics were applied to evaluate the interaction between four variables (fecal volume, composition, place and sex of coatis). A significant interaction between the parks and sexes with regard to volume and food category was not found. Ungrouped analysis allowed for the identification of a decreasing gradient in volume from PNC males, followed by PNC females, PMM males, and PMM females. We did not find differences between categories of food between males and females from PNC and PMM, except for invertebrates. Females from PNC consumed more invertebrates than males and females of PMM, but we did not find differences from PNC males. The coatis of both parks primarily consume invertebrates and vegetables, but garbage residues were found in their feces. Garbage fragments, such as paper, glass, metal, plastic and rope, cause a risk to the health, compromising the conservation efforts of wild coatis. Actions are needed to prevent the access of coatis to dumps in both parks.

## 1. Introduction

Sustainable ecotourism is an important strategy used in conservation areas to preserve and protect the environment while promoting economic development [1]. In addition to having political, ecological, and economic aspects, sustainable ecotourism also promotes greater interaction between humans and the environment, especially by increasing contact with local fauna and flora. This is a significant factor in raising awareness of the importance of preserving and conserving these environmental areas. However, it has now become a challenge simultaneously to meet the demands of ecotourism and the needs of conservation [2]. Non-consumptive uses of wildlife are increasing considerably [3,4], and negative impacts of ecotourism on different species of wildlife have already been reported [5,6]. There is, therefore, a growing concern with the management of visitors in conservation areas due to the trade-off between conservation and ecotourism [7,8].

Food remains taken into parks by tourists are often discarded in containers and places where it is impossible to avoid contact with the local fauna. Facilitated access to human-derived food resources has contributed to some wild animals adapting to and exploiting these resources [9]. It is widely accepted that wild animals should not have access to food discarded by tourists, due to the high risk of contamination by toxins, pathogens, and the ingestion of packaging fragments [10,11]. Depending on the volume, the type of food, and the opportunity of access, wild animals’ diets can change, changing the ecology of the species and, consequently, the ecology of other species in the region where they live [9]. Therefore, the continued use of discarded food by wild animals can have dramatic consequences on wild animal preservation.

Some mammalian species appear to be more adaptable to human coexistence due to feeding behavior and habituation, such as coatis [9,11,12,13], exploring human food sources. This supply of human food can be involuntary, due to poor garbage collection and containment in places visited by tourists or in houses near forested areas [11,12,13,14,15]. However, this adaptation has led to a considerable increase in the exploitation of human-derived food and materials unfit for consumption, which can directly affect the animals’ behavior and health, and indirectly affect their role in the ecosystem.

Coatis (*Nasua*: Linnaeus, 1766) are carnivores of the family Procyonidae, widely distributed in biomes such as the Atlantic Forest, Amazon Forest, Cerrados, Pantanal, and Chaco. Among the members of the Procyonidea family, the coatis are the most sociable, living in groups of females and their offspring [11,12,13]. When males reach their reproductive age, they abandon their groups and adopt solitary habits [11,12,13]. Coatis have an omnivorous diet, forage on the forest floor, and are highly opportunistic. However, some articles and anecdotal reports record that coatis are often observed feeding in the backyards of nearby houses or in park dumps [13,16,17].

In two ecological parks in Brazil, the Caparaó National Park (PNC) and the Municipal Park of Mangabeiras (PMM), the exploitation of discarded food sources by wild coatis has frequently been observed [16,17]. The parks differ in flow and ecophysiognomies, but in both, the activity of coatis foraging food from the trash is so ubiquitous that it has become a tourist attraction. On the other hand, some tourists claim a disturbance of leisure activities due to the proximity of the coatis. There is a great concern among park officials that the exploitation of human food may disrupt the feeding ecology of coatis. There is little knowledge about the food ecology of coatis in these conditions of proximity and its relative dependence on visitor activity in parks. Research on the type and volume of food consumed by the coatis in the two parks can help to understand the food ecology of a species that is still scarcely understood in its natural environment. Consequently, the development of educational measures that raise awareness about the ecological importance of coatis, as well as the proper disposal of garbage, is proposed.

Due to different metabolic needs, the exploitation of food items may also depend on the gender of the individuals [18]. Thus, an interaction between locations with potentially different available food resources and an individual’s sex can lead to different profiles in food consumption by wild coatis. For this reason, the fecal content of wild coati individuals that fed on items discarded by tourists in the PNC and PMM was investigated. The specific objectives were to compare (i) the coati diets in two ecological parks where there is ecotourism; (ii) whether these impacts may be contingent on the sex of the animals; (iii) and the implications of waste consumption on coatis feeding ecology. In general, this study contributes to a better understanding of the relationship between conservation areas and ecotourism, to make it possible to develop measures to improve the balance between these two activities.

## 2. Methods

### 2.1. Parks Studied

The study was conducted at two parks: Parque Municipal das Mangabeiras (PMM) and Parque Nacional do Caparaó (PNC). PMM is located on the southern boundary of the city of Belo Horizonte in the state of Minas Gerais (19°56′ S and 43°54′ W), Brazil. PMM has an area of 337 ha, with altitudes ranging from 300 to 1300 m. PMM is surrounded by areas of intense iron (Fe) mining activity. The park has a vegetation typology representative of the Cerrado biome, such as high-altitude fields, gallery forests, and proper *cerrado* itself [17]. Visits are free, on a day-use basis, with people able to picnic, practice sports, or walk along well-marked trails [17]. PMM’s estimated 360,000 visitors per year are diverse, including students, families, and friends who enjoy recreational activities in the concentrated core area. To support visitors, there are dumpsters, toilets, and tables. There are also some kiosks, which sell soda, beer, snacks, and sandwiches.

PNC is located between latitude 20°19′ S and 20°37′ S and longitude 41°43′ W and 41°53′ W, with a total area of 31,800 ha, containing many valleys, mountains, and river springs. Most of the park’s area is above 1300 m of altitude, with a Cwb-type climate (according to Koppen) that depends on the topographical types, with temperatures below zero at night [16]. The park’s vegetation is diverse, classified into four morphotypes: Coastal Evergreen Hygrophilous Forest, Tropical Subcaduciferous Forest, Coastal Vegetation, and High Altitude Fields. PNC is concerned with preservation and adventure, received 67,000 visitors in 2012 and 2013, and has four camping areas, which contain dumpsters, toilets, dishwashers, tables, benches, and barbecues [16].

### 2.2. Collection and Processing of Samples

The collection of feces was carried out in May 2012 in PMM, and in April and June 2012 in PNC. The fecal samples were collected directly from traps or park trails. Only adult coatis were captured in Tomahawk-like traps during the daylight, which were placed next to visitors’ trails and around areas where trash dumps were located. Each trap was baited with pieces of banana. Soon after the capture, veterinarians anesthetized the animals by injecting a solution of ketamine (10 mg/kg) and xylazine (0.5 mg/kg). Coatis frequently defecate when captured. The feces were then collected manually with the aid of gloves and tweezers when found in the traps. The identification of the sex of the coatis was possible from the samples collected in the traps. Other fresh feces were also collected along the coatis’ displacement trails and around the main visitation areas. Soon after collection, samples were identified and stored in bottles for analysis in the laboratory.

For each sample, the volume of feces was measured using a graduated beaker, with the displacement of the water column indicating the volume. The samples were then washed in running water through 4, 2, and 1 mm mesh sieves, placed in Petri dishes and taken to dry in an oven at a temperature of 25 °C. Subsequently, the samples were screened with the aid of a stereoscopic magnifying glass (40×) and fine-tipped tweezers.

The items were separated into 21 types (see *Data analysis*). To determine their importance in the coatis’ diet, the percentage of occurrence (PO) for each item was calculated by dividing its total frequency by the sum of the frequencies of all other elements [19], multiplied by one hundred (100). The results can, therefore, be presented as average percentages of the classification categories adopted in this study.

To complement this, behavioral data of the coatis were collected using the ad libitum method [20]. Informal interviews with park staff and residents of the surrounding area were also conducted in order to obtain information on the natural history of the coatis in those regions. The study was licensed by the Committee of Animal Use at the Federal University of Viçosa (n°. 03-2013) and by SISBio/ICMBio n° 31120-2/39017-1.

### 2.3. Data Analysis

In the analyses reported here, we included only one sample per individual and only used samples from adults of known sex. All analyses were conducted using R 4.0.0 [21]. The fit of all models was checked by evaluating residuals distributions in Q–Q plots, histograms, and boxplots. To evaluate the total volume and composition of feces between female and male coatis in the two parks (PMM and PNC), a GLM with Gaussian error distribution was used followed by the likelihood–ratio test (LRT) using the “stats” [21] and “car” packages [22], respectively. Individuals whose sex was not identified were removed from this analysis. In the factorial model, volume was added as a response variable, and the interaction between place (PMM or PNC) and sex (female or male) as a predictor variable (place*sex).

The age–sex class definition was based on the external morphology, body biometrics, tooth eruption, wear and staining of dentition, reproductive conditions, and weight [23]. To verify whether the composition of feces is different between females and males in the two parks, the food was grouped into five categories as follows: garbage (paper, onion skin, thread, plastic, aluminum, metallic debris, glass, and rubber), vertebrates (hair, bones, scales, feather, egg endosperm, and eggshell), invertebrates (larvae and arthropods), vegetal (seed, fruit, vegetables, and wood), and unidentified foods. From the analyses, it was possible to identify the effects of some food categories, providing a more complete view of anthropic impacts on coati diets at the two locations and for both sexes. Non-metric multidimensional scaling (NMDS) was used for this, followed by the analysis of similarity (ANOSIM) test with 999 permutations and Bray–Curtis distance, using the “vegan” package [24] for the analyses. Multiple ANOSIM tests were run posteriori. Then, to verify the contribution of individual food categories to the overall Bray–Curtis dissimilarity, similarity percentage (SIMPER) analysis was used.

Finally, to compare the percentage food category ingested between female and male coatis in the PMM and PNC parks, a GLM with beta error distribution was used, followed by LRT using the “glmmTMB” [25] and car packages, respectively. For this analysis, individuals whose sex was not identified were removed. In the factorial model, the percentage of occurrence of each food category was added as the response variable and the interaction between sex (female or male) and place (PMM or PNC) as the predictor variable (place*sex).

## 3. Results

In total, 99 fecal samples were evaluated, of which 44 were from females and 12 were from males (56 total samples from coatis’ with sex determined). It was not possible to identify the sex of the coatis’ who produced the other 43 samples. Of the 99 fecal samples obtained, 24 were from PMM, and 75 were from PNC. Considering that the analysis of fecal residues represents the diet of animals [19], the analyzed items were assumed to represent the diet of the coatis. Therefore, in the following paragraphs, items found in feces are considered food items ingested by the coatis.

**Figure 1:**
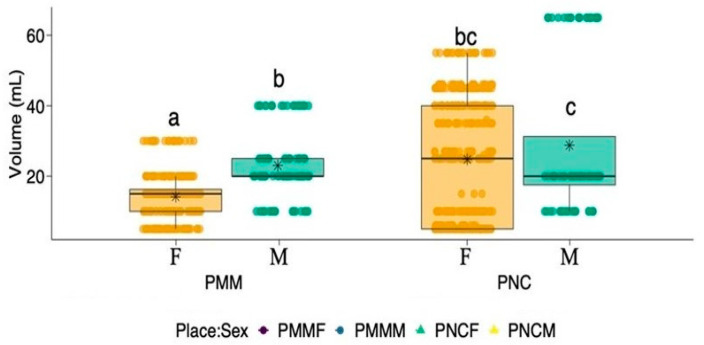
Variation in the fecal volume between female and male coatis (*Nasua nasua*) in two different parks, PMM (Parque Municipal Mangabeiras) and PCN (Parque Nacional do Caparaó). Letters represent statistical differences between groups by Estimated Marginal Means. F—Female, M—Male. Boxplots are represented by first and third quartiles, median, and mean (*). PMMF, female coati from Parque Municipal Mangabeiras; PMMM, male coati from Parque Municipal Mangabeiras; PNCF, female coati from Parque Nacional do Caparaó; PNCM, male coati from Parque Nacional do Caparaó.

Considering food categories (garbage, vertebrates, invertebrates, vegetation, and unidentified) as a response variable (grouped analysis), a significant interaction between the effects of parks and sexes on the total volume of food in the feces (LRT: χ^2^ = 3.961, df = 1, *p* < 0.05; Figure 1) was found. Simple main effects analysis also showed that the total volume of food was significantly influenced by both park (LRT: χ^2^ = 83.990, df = 1, *p* < 0.001; Figure 1) and sex (LRT: χ^2^ = 28.831, df = 1, *p* < 0.001; Figure 1). Coatis in PNC ingested a higher volume of food, and males ingested a higher volume of food than females. Overall, male coatis in PNC ingested the highest volume of food (28.7 ±21.4 mL), followed by females in PNC (24.7 ± 17.2 mL), males in PMM (23.0 ± 9.84 mL), and females in PMM (14.1 ± 6.73 mL).

**Figure 2:**
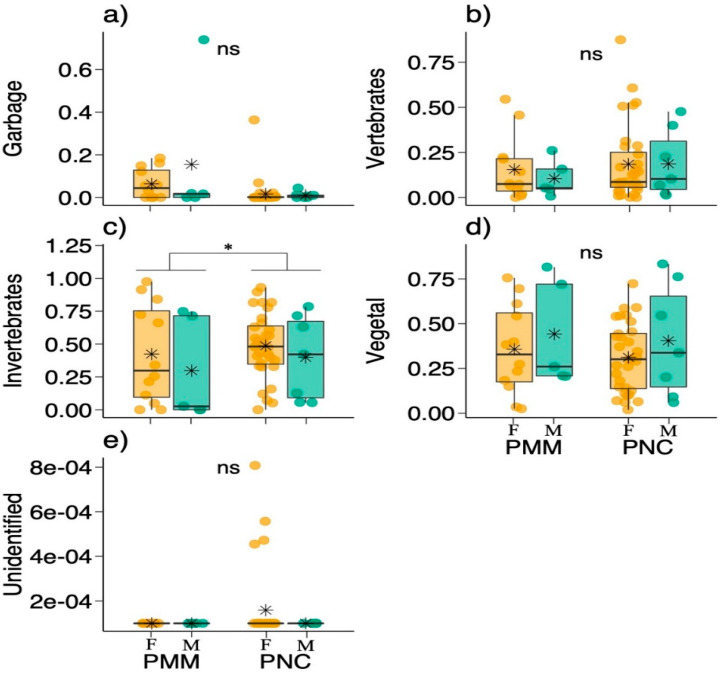
Variation in the percentage occurrence of food categories between female and male coatis (*Nasua nasua*) in two different parks, PMM (Parque Municipal Mangabeiras) and PCN (Parque Nacional do Caparaó). (**a**) Comparison of garbage consumption between males and females from both parks, PMM and PNC. (**b**) Comparison of vertebrate consumption between males and fe-males from both parks, PMM and PNC. (**c**) Comparison of invertebrate consumption between males and females from both parks, PMM and PNC. (**d**) Comparison of vegtal consumption be-tween males and females from both parks, PMM and PNC. (**e**) Comparison of unidentified mate-rial consumption between males and females from both parks, PMM and PNC. ns—non-significant. Asterisk at the top of panel (**c**) represents a significant difference between parks (*p* < 0.05). Boxplots are represented by first and third quartiles, median, and mean (*). Statistical results are depicted in Table 2. F—Female, M—Male. Asterisks on the boxplots represent the mean. PMMF, female coati from Parque Municipal Mangabeiras; PMMM, male coati from Parque Municipal Mangabeiras; PNCF, female coati from Parque Nacional do Caparaó; PNCM, male coati from Parque Nacional do Caparaó. Ns, no significant.

A significant interaction was found between the effects of parks and sexes on the food composition (ANOSIM R = 0.228; *p* = 0.003; Figure 2). The composition of food ingested by females in PNC was significantly different from that of both sexes in PMM (Figure 2). The food composition of males in PNC was similar to females in the same area, and to both sexes in PMM (Figure 2). Vegetation and invertebrates were the main food category contributors to differences in food composition between sexes in both parks (Table 1). The range of cumulative contribution from these two food categories was 30.7–54.2% for vegetation and 27.2–50.8% for invertebrates (Table 1). Vegetation and invertebrates contributed more than 73.5% to the difference in the composition of food ingested by females and males in the two parks (Table 1).

**Figure 3:**
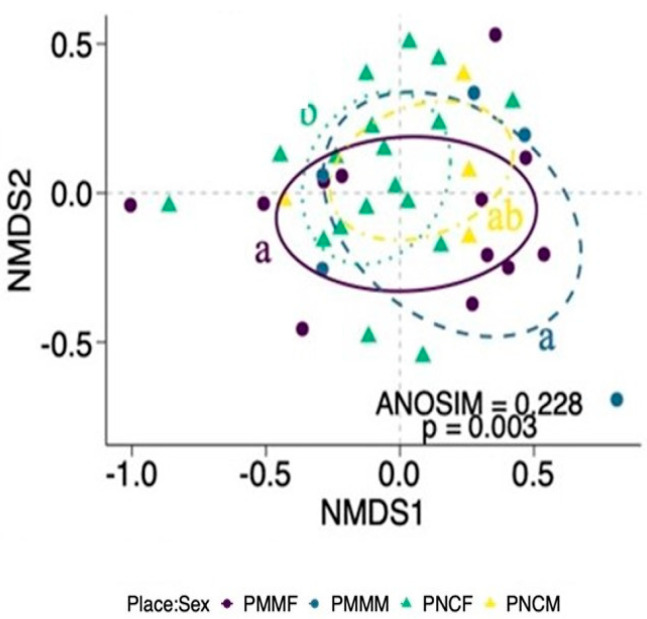
Variation in the food composition between female and male coatis (*Nasua nasua*) in two different parks, PMM and PCN. Letters represent statistical differences between groups by multiple ANOSIM tests. F—Female, M—Male. PMMF—PMM and female coati (solid line), PMMM—PMM and male coati (dashed line), PNCF—PNC and female coati (dotted line), PNCM—PNC and male coati (dashed–dotted line). PMMF, female coati from Parque Municipal Mangabeiras; PMMM, male coati from Parque Mu-nicipal Mangabeiras; PNCF, female coati from Parque Nacional do Caparaó; PNCM, male coati from Parque Nacional do Caparaó.

No significant interaction was found between the effects of parks and sexes on the percentage occurrence of any food category (Figure 3; Table 2). Simple main effects analysis also did not show any influence of parks or sexes on the percentage occurrence of food categories, except for invertebrates, in which the coatis from PNC ingested significantly more invertebrates than the coatis from PMM (Figure 2; Table 2). Considering food categories, the main categories ingested by coatis in the two parks, regardless of sex, were invertebrates and vegetation (Table 1). These two categories make up, on average, 73.9–80.2% of the coati diet. Furthermore, garbage can reach, on average, 15.4% of the diet of some coatis.

## 4. Discussion

Two different coati feeding ecology profiles were found in the two parks, with some emergence of sex differences. Coatis from PNC ingested a higher volume of food than coatis from PMM, and males tended to ingest higher volumes of food than females. The food composition was significantly different between the sexes in the two parks. Finally, while the two most frequently ingested food types were vegetation and invertebrates, debris from garbage was detected in a high percentage in fecal samples from both sexes in both parks. Overall, it was observed that conservation areas, which promote ecotourism directly, affect the diet of wild coatis, with the parks and the sex of the animal directly influencing the volume and composition of the food ingested.

The volume of feces of carnivores is the result of the amount of food ingested and the non-digestible material in healthy animals [26]. We assume that the coatis in the PNC have a smaller amount of food items of human origin, which are more digestible and caloric [27]. In PMM, coatis have contact with residues from a visitor population approximately five times greater than in PNC. Therefore, we argue that PNC coatis search for food with more calories and ingest food with a higher non-digestible polymer content, such as cellulose and chitin. The higher energy requirement and the indigestible material result in a greater volume of feces in the PNC coatis compared to the PMM coatis.

The difference between the sexes within each park may be the result of the smaller size of the females, which eat less food. However, the consumption of arthropods was higher in females from the PNC compared to males, suggesting that there is an intersexual niche separation. Our results are intriguing, as they converge with different food niches between female and male coatis, which is believed to be a force that determines the social structure of multiple females and offspring, and solitary adult males [28]. The difference between sexes that we found in our study is similar to that of other mammal species, in which females develop strategies to consume foods with a higher energy content and digestibility (e.g., in *Cervus elaphus*; in primates) [29,30].

The sub-population from PNC ingests more invertebrates and insects than the coatis from PMM. This higher invertebrate intake seems to be the consequence of a higher intake of insects by females in PNC, when compared to the males from the same park and both males and females from PMM. The balance between greater nutrient/energy demand and food availability in each park may explain the differences in the food consumption of the two sub-populations.

The PNC is a mountainous region surrounding the third highest peak in Brazil (Pico da Bandeira), with many valleys and steep mountain slopes. Although located in a tropical area, the climate in PNC has large thermal fluctuations, and temperatures can fall below zero at night [16]. The coatis from PNC in this study use paths close to the visitors’ trails, moving from the base of the trail to higher areas, gaining an estimated 1600 m of elevation over a 4.1 km distance (personal observation). Despite the long-distance and highest altitude, coatis from PNC can be observed on one day at the base and on the next day near the peak. The effort and energy expenditure of the coatis to move in these conditions in the PNC seems to be much greater than that of the coatis that live in the PMM.

The movement of coatis in PNC contrasts sharply with that of the coatis in PMM, which were observed on a daily basis around the core area of the park, where recreation activities are located. The walking trails for visitors in PMM are short, with lengths between 1000 and 1300 m, without a significant altitude gradient. The coatis in PMM move along the trails after tourists but are concentrated during most of the day in the visitors’ recreation area. The number of visitors in PMM is 5.4 times higher than that in PNC, which should generate more available food waste for coatis. In addition, the average temperature in PMM is 21.1 °C and never approaches negative values [17].

It is not only the volume that differentiates the two parks but the content of sugars and fats that make up the processed foods that are consumed by tourists. Brazilian eating habits include many processed foods, which are rich in carbohydrates and fats [27]. Recreational activities in PNC and PMM include the consumption of processed foods by visitors, which are ready-to-eat and non-perishable. These foods are attractive to coatis and serve as a supplemental source of energy. The metabolic energy expenditure for coatis from PMM seems to be lower than that for coatis from PNC, as there are shorter distances to be covered with concentrated food resources and a milder climate. The greater availability of discarded food is an easily accessible energy source for PMM coatis. On the contrary, the availability and dispersion of food sources are lower for PNC coatis, which results in higher energy expenditure to obtain discarded foods that are mostly processed.

Contrasting with their low carbohydrate content (2–10%), insects are a rich source of protein (20–70%), amino acids (30–60%), and fat (10–50%) [31]. For most mammals, especially females, amino acids are rich sources to meet physiological needs for proteins, enzymes, hormones, and immune defense molecules [32]. The metabolic demand is greater in environments where coatis have to traverse larger areas with a high altitude gradient [33], requiring not only physical energy but also energy for other metabolic systems, such as reproduction, which is known to be a higher cost for females [34].

The abundance and diversity of insects were not assessed, but the location of the two parks can have an influence on the availability of invertebrates. The decline in the diversity and abundance of insects is occurring on all continents [35]. There are no studies indicating a reduction in insect diversity in the two parks studied, but there is evidence in the literature that urban parks, such as PMM, are those most affected by environmental changes, nocturnal light, and the use of insecticides [35]. PMM lies alongside the metropolitan region of Belo Horizonte, which has 5.9 million inhabitants. In contrast, only one small town (5.257 habitants), coffee plantations, and small cattle ranches surround PNC. The area of PNC is 94.4 times larger than that of PMM, with vegetation classified into four morphotypes, with much greater biodiversity [16]. Therefore, PNC should be expected to have greater diversity and abundance of insects and other invertebrates, which coatis can use as their most easily available food source.

PNC females seem to make use of more valuable food items to supply energy and specific nutrient needs. PNC males consume a greater volume of food than females, as do coatis of both sexes in the PMM. This suggests that females have adopted a more selective food consumption strategy than males, as has been observed in other mammals [26]. This strategy is advantageous to females that have to expend greater energy in the reproduction, lactation, and defense of their offspring.

A lower intake of invertebrates and insects by PMM females compared to PNC females may be related not only to the lower availability of insects but also to the greater availability of human food discarded in PMM. An abundant and predictable supply of discarded food can lead to a lower intake of insects, which are an unpredictable food source [36]. This effect was also observed in PMM males compared to PNC males, suggesting that the lower abundance of insects can be counterbalanced with greater availability of discarded food.

Garbage debris, such as paper, glass, plastic, and metal, are ingested by coatis in the two parks, regardless of sex. These materials substances are foreign bodies, which can potentially cause severe disease, resulting in the erosion of the gastric mucosa, obstruction and intoxication [37]. Therefore, the ingestion of garbage debris can be highly risky and damage the teeth and digestive system. The percentage of garbage debris in the diet of some coatis in both parks can reach unacceptably high levels. Data on the mortality of coatis caused by garbage debris were not found in the scientific literature, but severe damage to wild animals was recorded in many studies [2,5,6,7,8,9,10]. The ingestion of garbage by coatis is frequent and has been recorded in many studies. For example, a study recorded the presence of 60% of household foods in fecal samples of wild coatis living in an urban woodland [13]. In another study conducted at PMM, between 1995 and 1998, 9.7% organic residues were found in wild coatis’ feces [11]. In addition, domestic paper trash was recorded in the stomach contents of 23 road-killed coatis [38]. Our results are in accordance with the garbage exploration by coatis in parks or nearby houses, which warrants more attention in the conservation efforts for this species.

In general, the results from this study agree with those of other studies on the feeding ecology of coatis, which recorded a diet based on vegetal material and invertebrates [11,12,13,38,39,40]. As reported in other studies, this investigation characterizes coatis as highly opportunistic carnivores that regularly adapt to the exploitation of food sources that have been discarded by people. Nonetheless, other unmeasured variables, such as insect abundance, size of individuals, and the daily range of the coatis, can contribute to the number of insects in the feces of PNC coatis.

Despite the great effort to obtain samples in difficult logistic conditions, our study has some sample limitations. First, our sample is relatively small, despite representing two populations of coatis. Second, our sampling refers to feces collected close to the visitation sites, not including other groups of coatis that have no contact with humans. Third, our collection was restricted to two months of a year only. In the PNC, tourism is seasonal and is restricted in rainy months due to the risk of ascending to the higher parts of the park [16]. Therefore, it is plausible to expect seasonal changes in the diets of coatis, due to both climatic variations and the number of tourists. For example, we have unsystematic records that PNC coatis “invade” coffee plantations and orchards near the city to forage in periods with low tourist flow. Finally, highly digestible items are not observable through a stereoscope in the fecal examination and, consequently, were not observed in this study. For this reason, it should be considered as a general characterization of the type of diet in an environment with ecological tourism, where visitors leave behind poorly conditioned food residues.

## 5. Conclusions

In this study, location- and sex-dependent differences were not found, which suggests that other non-combined pressures determine the presence of food items in feces. Arthropods seem to be the food source that differentiates the coatis of PNC from the coatis of PMM. The higher demand for energy and nutrients by PNC female coatis seems to contribute to the greater number of arthropods found in their feces. The involuntary inclusion of garbage into the diet of wild coatis caused by visitors is a risk to their health and compromises preservation efforts for wild coatis. It is necessary to investigate the effect of garbage consumption on metabolism, lesions in the digestive system and possible pathogens that can be transmitted by fomites (cans, plastic and other objects from garbage). The investigation must include other groups of coatis from the two parks that do not have contact with visitation areas.

Long-term studies could elucidate the impact of waste consumption on feeding ecology, social behavior and population dynamics. Preventatively, simple and efficient measures should be adopted, such as a permanent environmental education program for visitors to avoid the misuse of garbage. It will also be necessary to increase the efficiency of disposal devices for food and packaging, preventing wild coatis from having access to this material. As a whole, this study contributes to the understanding of the feeding ecology of coatis in relation to contact with visitors in ecological parks.

## Figures and Tables

**Table 1:** Contrast tables of the contribution of individual food categories (garbage, vertebrates, invertebrates, vegetal, and unidentified) to the overall Bray–Curtis dissimilarity of the composition of food ingested by females and males of coatis (*Nasua nasua*) in two different parks (PMM and PNC). PMMF—PMM and female coati, PMMM—PMM and male coati, PNCF—PNC and female coati, PNCM—PNC and male coati. PMM, Parque Municipal Mangabeiras; PNC, Parque Nacional do Caparaó; PMMF, female coati from Parque Municipal Mangabeiras; PMMM, male coati from Parque Municipal Mangabeiras; PNCF, female coati from Parque Nacional do Caparaó; PNCM, male coati from Parque Nacional do Caparaó. Av.dissim, average dissimilarity; SD, standard deviation. NO, unidentified. Contrib., contribution.

**PMMF_PMMM**	**Av. Dissim**	**SD**	**Average Female**	**Average Male**	**Cumulative**	**Cumulative %**	**Contrib. %**
Invertebrates	0.30	0.26	258.67	95.20	0.43	42.79	42.79
Vegetal	0.21	0.21	57.00	145.40	0.73	73.48	30.69
Garbage	0.11	0.19	17.50	48.00	0.89	88.84	15.36
Vertebrates	0.08	0.10	37.92	29.60	1.00	100.00	11.16
NI	0.00	0.00	0.00	0.00	1.00	100.00	0.00
**PMMF_PNCF**	**Av. Dissim**	**SD**	**Average Female**	**Average Male**	**Cumulative**	**Cumulative %**	**Contrib. %**
Invertebrates	0.35	0.22	258.67	374.11	0.51	50.78	50.78
Vegetal	0.21	0.18	57.00	314.83	0.82	81.96	31.18
Vertebrates	0.10	0.11	37.92	95.61	0.97	97.26	15.29
Garbage	0.02	0.03	17.50	5.56	1.00	99.99	2.74
NI	0.00	0.00	0.00	0.11	1.00	100.00	0.01
**PMMF_PNCM**	**Av. Dissim**	**SD**	**Average Female**	**Average Male**	**Cumulative**	**Cumulative %**	**Contrib. %**
Vegetal	0.35	0.28	57.00	437.75	0.50	49.96	49.96
Invertebrates	0.25	0.27	258.67	108.00	0.86	86.10	36.14
Vertebrates	0.07	0.09	37.92	50.50	0.96	96.29	10.19
Garbage	0.03	0.03	17.50	14.00	1.00	100.00	3.71
NI	0.00	0.00	0.00	0.00	1.00	100.00	0.00
**PMMM_PNCF**	**Av. Dissim**	**SD**	**Average Female**	**Average Male**	**Cumulative**	**Cumulative %**	**Contrib. %**
Invertebrates	0.29	0.17	95.20	374.11	0.45	45.02	45.02
Vegetal	0.22	0.17	145.40	314.83	0.79	78.85	33.83
Vertebrates	0.08	0.08	29.60	95.61	0.91	90.98	12.12
Garbage	0.06	0.12	48.00	5.56	1.00	99.99	9.01
NI	0.00	0.00	0.00	0.11	1.00	100.00	0.01
**PMMM_PNCM**	**Av. Dissim**	**SD**	**Average Female**	**Average Male**	**Cumulative**	**Cumulative %**	**Contrib. %**
Vegetal	0.37	0.23	145.40	437.75	0.54	54.22	54.22
Invertebrates	0.19	0.19	95.20	108.00	0.82	81.50	27.29
Garbage	0.07	0.16	48.00	14.00	0.92	92.47	10.97
Vertebrates	0.05	0.04	29.60	50.50	1.00	100.00	7.53
NI	0.00	0.00	0.00	0.00	1.00	100.00	0.00
**PNCF_PNCM**	**Av. Dissim**	**SD**	**Average Female**	**Average Male**	**Cumulative**	**Cumulative %**	**Contrib. %**
Vegetal	0.30	0.22	314.83	437.75	0.49	49.21	49.21
Invertebrates	0.23	0.20	374.11	108.00	0.87	86.92	37.70
Vertebrates	0.07	0.08	95.61	50.50	0.98	98.25	11.33
Garbage	0.01	0.01	5.56	14.00	1.00	99.99	1.75
NI	0.00	0.00	0.11	0.00	1.00	100.00	0.01

**Table 2:** Likelihood-ratio tests comparing the percentage occurrence of each food category ingested by female and male coatis (*Nasua nasua*) in two different parks (PMM and PNC). Sex—male and female; Place—PMM and PNC. * Significant *p*-value.

Response	LRT	Df	*p*-Value
**Garbage**			
Sex	0.065	1	0.798
Place	1.724	1	0.189
Sex:Place	0.023	1	0.879
**Vertebrates**			
Sex	0.043	1	0.836
Place	0.686	1	0.407
Sex:Place	0.012	1	0.913
**Invertebrates**			
Sex	1.717	1	0.190
Place	4.635	1	0.031 *
Sex:Place	0.757	1	0.384
**Vegetation**			
Sex	1.497	1	0.221
Place	0.235	1	0.628
Sex:Place	0.069	1	0.792
**Unidentified**			
Sex	0.426	1	0.514
Place	0.794	1	0.373
Sex:Place	0.255	1	0.614
